# Sequencing and Analysis of the Mitochondrial Genome of *Aedes aegypti* (Diptera: Culicidae) from the Brazilian Amazon Region

**DOI:** 10.3390/insects14120938

**Published:** 2023-12-11

**Authors:** Andrelina Alves de Sousa, Ana Cecília Ribeiro Cruz, Fábio Silva da Silva, Sandro Patroca da Silva, Joaquim Pinto Nunes Neto, Maria Claudene Barros, Elmary da Costa Fraga, Iracilda Sampaio

**Affiliations:** 1 Post-Graduate Program in Genetics and Molecular Biology, Federal University of Pará, Belém 66075-110, Pará, Brazil; andrelbio@yahoo.com.br; 2 Evandro Chagas Institute (IEC/SVS/MS), Department of Arbovirology and Hemorrhagic Fevers, Ananindeua 67030-000, Pará, Brazil; anacecilia@iec.gov.br (A.C.R.C.); fabiodasilva@iec.gov.br (F.S.d.S.); sandrosilva@iec.gov.br (S.P.d.S.); joaquimneto@iec.gov.br (J.P.N.N.); 3 Post-Graduate Program in Parasite Biology in the Amazon, Center of Biological and Health Sciences, Pará State University, Belém 66095-662, Pará, Brazil; 4 Laboratory of Genetics and Molecular Biology (GENBIMOL), Maranhão State University, Caxias 65604-380, Maranhão, Brazil; mbdene@yahoo.com.br (M.C.B.); elmaryfraga@yahoo.com.br (E.d.C.F.); 5 Laboratory of Evolution, Institute of Coastal Studies, Federal University of Pará, Bragança 68600-000, Pará, Brazil

**Keywords:** Legal Amazon, Culicidae, mtDNA, phylogeny, population genetics

## Abstract

**Simple Summary:**

Responsible for the transmission of arboviruses, the mosquito *Aedes aegypti* is a major challenge to public health in Brazil, particularly in urban areas located within tropical and subtropical regions. The recent epidemiological bulletins from the Brazilian Health Ministry in the state of Maranhão have reported high levels of urban infestation by this vector, which represents a potential risk for the occurrence of new epidemics. In this context, the present study describes the partial mitochondrial genome of a sample of *Ae. aegypti* from the Brazilian state of Maranhão. Studies of this type provide important insights into the biological evolution of this important species and have the potential to contribute to the development of increasingly effective strategies for the control of this disease vector.

**Abstract:**

*Aedes aegypti* is a mosquito native to the African continent, which is now widespread in the tropical and subtropical regions of the world. In many regions, it represents a major challenge to public health, given its role in the cycle of transmission of important arboviruses, such as Dengue, Zika, and Chikungunya. Considering the epidemiological importance of *Ae. aegypti*, the present study sequenced the partial mitochondrial genome of a sample collected in the municipality of Balsas, in the Brazilian state of Maranhão, followed by High Throughput Sequencing and phylogenetic analyses. The mitochondrial sequence obtained here was 15,863 bp long, and contained 37 functional subunits (thirteen PCGs, twenty-two tRNAs and two rRNAs) in addition to a partial final portion rich in A+T. The data obtained here contribute to the enrichment of our knowledge of the taxonomy and evolutionary biology of this prominent disease vector. These findings represent an important advancement in the understanding of the characteristics of the populations of northeastern Brazil and provide valuable insights into the taxonomy and evolutionary biology of this prominent disease vector.

## 1. Introduction

Mosquitos (Diptera: Culicidae) have a virtually cosmopolitan distribution, particularly in the world’s temperate and tropical regions [1]. While the first mosquito species were described in the eighteenth century, the link between these insects and the transmission of infectious diseases, such as filariasis and malaria, was only discovered at the end of the nineteenth century. Almost two centuries after the first scientific studies of these organisms, approximately 3600 mosquito species have now been described formally, although many more species are assumed to exist, highlighting the need for extensive revision of the Culicidae and, in particular, studies that provide insights into the evolutionary drivers of the group [1].

In epidemiological terms, *Aedes* (*Stegomyia*) *aegypti* (Linnaeus, 1762) is one of the most important mosquito species, not least because of its intimate relationship with human populations, which has allowed it to spread throughout all the world’s continents, except Antarctica. The species is often targeted specifically in government campaigns for the control of disease vectors and is often associated with human migrations and unregulated urban development [2]. This mosquito is involved in the transmission cycle of a large number of viruses, including the principal arboviruses that affect human populations [3], that is, *Orthoflavivirus denguei* (DENV serotypes I, II, III and IV), *Orthoflavivirus flavi* (YFV), *Orthoflavivirus zikaense* (ZIKV), and *Chikungunya virus* (CHIKV).

*Aedes aegypti* has a complex evolutionary history, with studies indicating an initial major dispersal from Africa to the Americas, and, subsequently, to Asia, although there is also evidence of the parallel dispersal of two distinct lineages that originated in eastern and western Africa [4]. The species was almost certainly introduced into the Americas during the Age of Discovery, between the sixteenth and eighteenth centuries, when it expanded rapidly throughout most of tropical South and Central America [5,6].

In recent decades, a number of studies have investigated the genetic variability of *Ae. aegypti* populations, mainly using molecular markers extracted from regions of the nuclear [7,8] and mitochondrial genomes [5,9,10,11,12]. These studies have revealed significant differences among populations, which have led to the hypothesis that two principal *Ae. aegypti* lineages are found in Brazil, and worldwide. In this context, the mitochondrial (mtDNA) genome is an important tool for evolutionary studies, given its uniparental inheritance, lack of recombination and high mutation rates, in comparison with the nuclear genome [13,14,15,16]. Mitochondrial markers are especially useful for the study of vectors of medical and epidemiological importance [17,18,19,20,21,22,23,24,25].

The state of Maranhão is located in the northeast region of Brazil, and coincides with the transition zone between the Amazon, Cerrado and Caatinga biomes. Recent epidemiological bulletins of the Brazilian Health Ministry, Maranhão have revealed high levels of urban infestation by *Ae. aegypti*, which represents a potential risk for public health. Given the epidemiological importance of this vector and its high population densities in the state, it is necessary to understand the genetic variability of *Ae. aegypti* and its population structure, in order to support the development of effective strategies of population control, adapted to the characteristics of the populations found in northeastern Brazil.

Given the global epidemiological importance of this species, the present study provides the first description of the mitochondrial genome of a representative sample of *Ae. aegypti* population from the state of Maranhão, in northeastern Brazil, using High Throughput Sequencing for the first time.

## 2. Materials and Methods

### 2.1. Collection of the Biological Samples and the Extraction of the Total DNA

**Figure 1:**
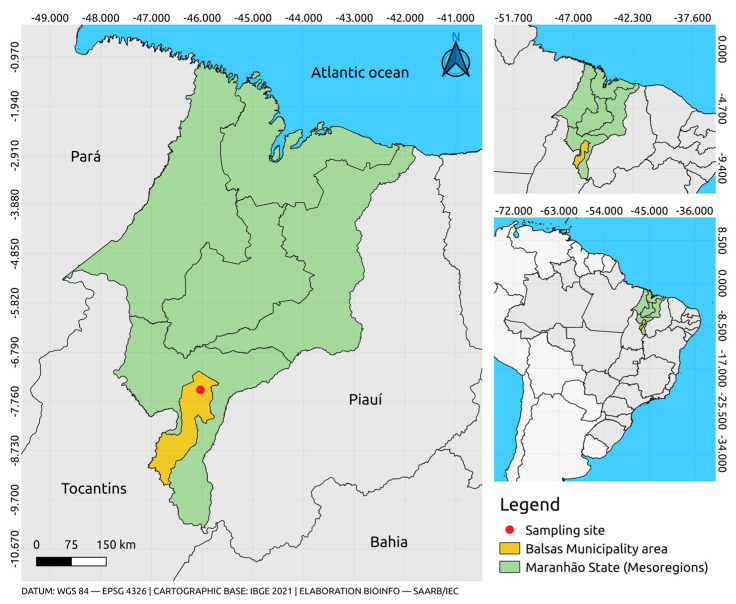
Location of the municipality of Balsas, in Maranhão state, showing the site from which the biological samples were collected.

The biological samples of *Ae. aegypti* analysed in the present study were collected during expeditions to the municipality of Balsas, in the state of Maranhão (−7.53′29.17″ S, −46.03′74.56″ W), in the mid–north region of Brazil (Figure 1). This region is a subdivision of the Brazilian Northeast, which coincides with the transition zone between the Amazon and Cerrado biomes, and also includes the western half of the state of Piauí [26]. The samples were collected using 30 ovitraps, which were distributed in different neighbourhoods of the municipality at intervals of at least 1 km. The traps were set in shady and poorly lit areas protected from the rain in the vicinity of residential infrastructure. The sites were selected previously during the rapid index surveys of *Ae. aegypti* (LIRAa) conducted in the municipality. The traps were retrieved after six days and transported to the Genetics and Molecular Biology Laboratory at the Caxias campus of Maranhão State University (UEMA).

The ovitraps contained small (12 cm × 2.8 cm) palettes on which the mosquitos laid their eggs during the trapping period. In the laboratory, the palettes were immersed in water to allow for the eclosion of the larvae, which were then kept in artificial nurseries until the emergence of the adults, which were identified using stereoscopic loupes and the dichotomous classification key, developed by Consoli and Lourenço-de-Oliveira [27], which is based on specific features of the external morphology of the adult specimens. Adults of the study species (*Ae. aegypti*) were transferred to entomological cages designed for breeding, where they were maintained with a 10% saccharose solution.

After two feeding cycles, a total of 20 females were isolated to lay eggs, which were isolated in individual artificial incubators until the eclosion of the larvae, which were fed until the fourth development stage (L4), following the protocol established by Santos et al. [28]. Once the larvae reached stage L4, they were transferred to 1.5 mL Eppendorf micro-tubes containing 70% alcohol as a preserver and stored in a freezer at −80 °C until the extraction of the total DNA.

The total DNA of each sample was extracted using the Wizard Genomic DNA Purification kit (Promega, Madison, WI, USA), following the manufacturer’s recommendations. The products extracted from each sample were quantified using a Qubit 2.0 fluorometer (Invitrogen, Waltham, MA, USA), together with a dsDNA Hs Assay kit (Invitrogen, Waltham, MA, USA).

### 2.2. Genomic Sequencing

The genomic sequencing, computer analyses and compilation of the results were all conducted at the Arbovirology and Hemorrhagic Fevers Section of the Evandro Chagas Institute (SAARB-IEC/MS/SVSA) in Ananindeua. The genomic library was compiled from a single sample, which had been quantified previously and standardised to a concentration of 0.2 ng/μL, and then fragmented and marked with two adapter sequences (i7 and i5) using the Nextera XT DNA Library Preparation kit (Illumina, San Diego, CA, USA) following the manufacturer’s protocol. The sample was then quantified using a Qubit 2.0 fluorometer (Invitrogen) and the fragment size was evaluated using a High Sensitivity DNA analysis kit (Agilent Technologies, Santa Clara, CA, USA) in a BioAnalyzer (Agilent Technologies). The final product was used for genomic sequencing using a NextSeq 500/550 High Output kit (Illumina) for 300 cycles (2 × 150) on a NextSeq 500 System platform.

### 2.3. Data Processing and Description of the Genome

The raw reads were initially evaluated qualitatively using Fastp v. 0.23.2 [29], configured to remove the adapter sequences, reads with a PHRED quality score of less than 20, and reads of less than 50 nt in length. To better isolate the reads that correspond to those of the study species, the data were mapped against a reference genome (*Aedes aegypti* L5.0, mtDNA GenBank ID: NC_035159) using a combination of Bowtie2 v.2.5.1 [30] e Samtools v.1.17 [31]. The filtered data were then used to mount the genome using the De Novo method. The contig was assembled using the MEGAHIT v.1.2.9 software [32], with the default mounting configurations (in k-mer lengths of 21, 29, 39, 59, 79, 99, 119 and 141 nt). The mitochondrial contigs obtained here was identified using the DIAMOND v.2.1.6.160 [33] in BlastX alignment mode, considering an e-value of 10^−5^, and inspected manually in Geneious v.11.1.5 [34].

The final mitochondrial sequence was annotated using the online MITOchondrial genome annotation Server tool (MITOS) [35] and fitted to a circular configuration, based on the identification of overlaps using Blastn v.2.14.0 [36], and was also used as a reference for the remapping of the mitochondrial reads in Bowtie2, to obtain their coverage metrics. The linear structure of the sequence was determined using CGview [37], and its composition and nucleotide parameters and the relative synonymous codon usage (RSCU) were obtained using MEGA X v.10.2.6 [38] and Geneious v.11.1.5 [34], respectively. Skews in the nucleotide composition were calculated using the formulas AT-skew = (A − T)/(A + T) and GC-skew = (G − C)/(G + C) [39]. To evaluate the selection pressure affecting the PCGs of the study species of the tribe Aedini, the ratios of non-synonymous (*dN*) to synonymous (*dS*) substitutions were calculated in CodeML, available in the PAML package [40]. All the graphs presented here were produced using the R software v.4.2.3 (available at: https://www.r-project.org accessed on 23 June 2023), together with the *ggplot2*, *reshape2* and *pheatmap* packages.

### 2.4. Phylogenetic Analysis

The phylogeny of the mosquitoes was reconstructed based on all 13 protein codifying regions of the *Ae. aegypti* sequence obtained here, together with those of the other 28 mtDNAs available in the public databases (Supplementary Table S1). The regions corresponding to each PCG of the target sequences were extracted in parts, aligned using MAFFT v.7.520 [41], inspected manually in Aliview v.1.28 [42], and concatenated using Seqkit v.2.4.0 [43]. The nucleotide distances between the study taxa were obtained using MEGA X (based on the maximum likelihood composition model). The phylogeny was reconstructed using the maximum likelihood method, with the prior definition of the best nucleotide substitution model (GTR+F+I+G4) based on the Akaike information criterion (AIC), using the IQ-TREE v.1.6.12 software [44], with the bootstrapping (BPP) set to 1000 repetitions. The topology obtained from these analyses was visualized in FigTree v.1.4.4 (available at: http://tree.bio.ed.ac.uk/software/figtree accessed on 26 June 2023) and edited with Inkscape v.0.92 (available at: https://inkscape.org/pt-br accessed on 30 June 2023).

## 3. Results

**Figure 2:**
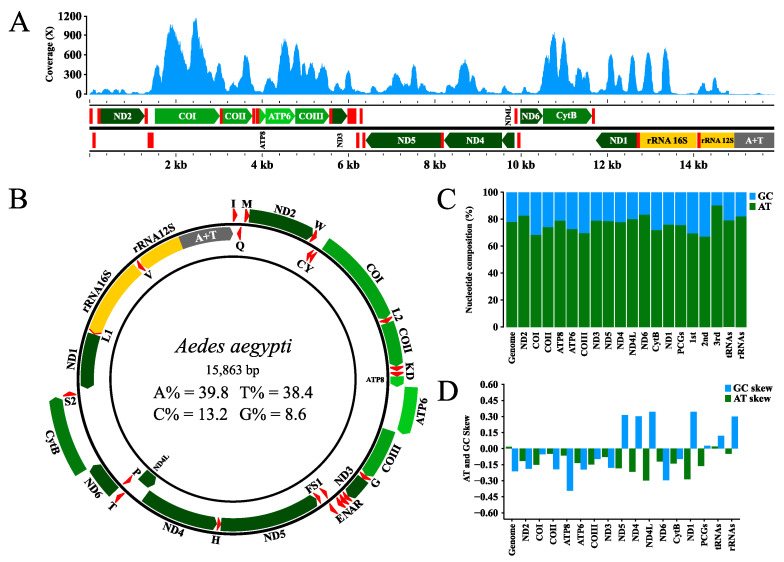
(**A**) Genomic coverage and structural organisation of the sequence obtained in the present study, showing the 37 functional subunits (13 PCGs, 22 tRNAs and 2 rRNAs), and the partial portion control region rich in adenine and thymine (A+T), (**B**) circular configuration of the genome, (**C**) AT% and GC% nucleotide composition and (**D**) AT/GC skews.

The genomic sequencing generated a total of 75.5 million reads, with 94% approval, following the quality control, and after the exclusion of the adapters and the bases with PHRED quality scores of less than 20. A contig of 15,863 bp was also obtained (Figure 2A,B) with a mean coverage depth of 217.4×, which corresponds to 28,539 of the total number of reads generated, being composed with 37 functional subunits (13 PCGs, 22 tRNAs and 2 rRNAs) with a partial portion of the control region rich in adenine and thymine (A+T) (Supplementary Table S2).

The overall AT content of the sequence obtained here is 78.2% (Figure 2C), with a generally positive AT skew (which indicates a greater adenine content in comparison with thymine) and a negative GC skew (indicating a relatively greater cytosine content versus guanine) (Supplementary Table S2), although with inversions, depending on the different arrangements of the subunits and regions evaluated (Figure 2D). An additional 22 small intergenic regions were also identified between the annotated subunits, ranging in length from 1 to 45 bp.

A typical secondary structure of the cloverleaf type was observed in 21 of the 22 tRNA subunits annotated here, which had lengths varying from 64 bp (tRNA*^Arg^*) to 72 bp (tRNA*^Val^*) with a mean AT content of 77.9% (Supplementary Table S2). This structure has four arms (amino acid acceptor (AA), dihydrouridine (DHU), TΨC and anticodon acceptor (AC)) and four loops (AA, DHU, TΨC and Variable (V)). The only variation was observed in the tRNA*^Ser1^* subunit, in which the dihydrouridine (DHU) arm has been substituted by a DHU loop (Supplementary Figure S1).

**Figure 3:**
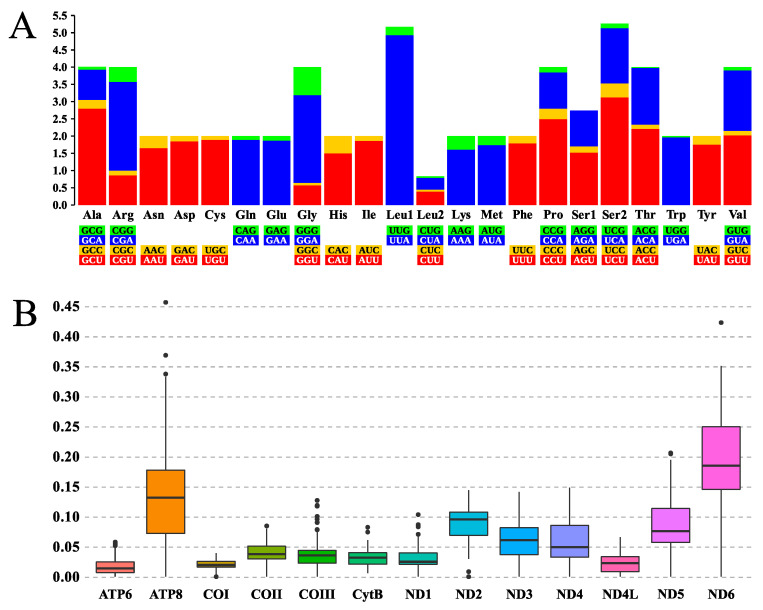
(**A**) Relative synonymous codon usage (RSCU) of the mtDNA of *Ae. aegypti*. The RSCU values are shown on the *y* axis, while the families of synonymous codons and their respective codifying triplets are shown on the *x* axis. (**B**) Boxplot of the mean evolutionary pressure that affects the PCGs identified in the present study, based on the ratio of non-synonymous (*dN*) to synonymous (*dS*) substitutions (*dN/dS*) recorded in the set of taxa closed related to the genus *Aedes*, including the sequence obtained here. The *dN/dS* ratios are shown on the y axis, and the PCGs, on the *x* axis.

The 13 PCGs annotated from the sequence obtained here vary in length from 159 (*ATP8*) to 1740 bp (*ND5*), with an AT content ranging from 72.5% (*ND2*) to 83.6% (*CytB*). The most frequent start codons were of two types (ATG and ATT), with complete stop codons of the TAA types. Excluding stop codons, a total of 3719 amino acid triplets were recorded, with a significant AT skew in the third position of the amino acids expressed in the codifying regions of the sequence. The relative synonymous codon usage (RSCU) indicates that only the triplet AGG (*tRNA^Ser1^*) was not present in the PCGs, and that a majority of the codons terminating with adenine or uracil (thymine) were expressed significantly (RSCU > 1) more often than those terminating with cytosine or guanine. Overall, the amino acid expressed most frequently in the sequence was UUA (*tRNA^Leu1^*), with 484 occurrences (RSCU = 4.93), while the least expressed was ACG (*tRNA^Tyr^*), with only a single occurrence, with RSCU = 0.02 (Figure 3A).

The evaluation of the parameters of the evolutionary pressures that influence the PCGs was based on the ratio of non-synonymous to synonymous substitutions (*dN*/*dS*) and the comparison of the sequence obtained here with those of other taxa related closely to the genus *Aedes*. This analysis indicated that the different codifying regions have evolved globally under the influence of purifying pressure (*dN/dS* > 1), with mean ratios ranging from 0.001 ± 0.0586 in *ATP6* to 0.001 ± 0.4573 in *ATP8* (Figure 3B).

**Figure 4:**
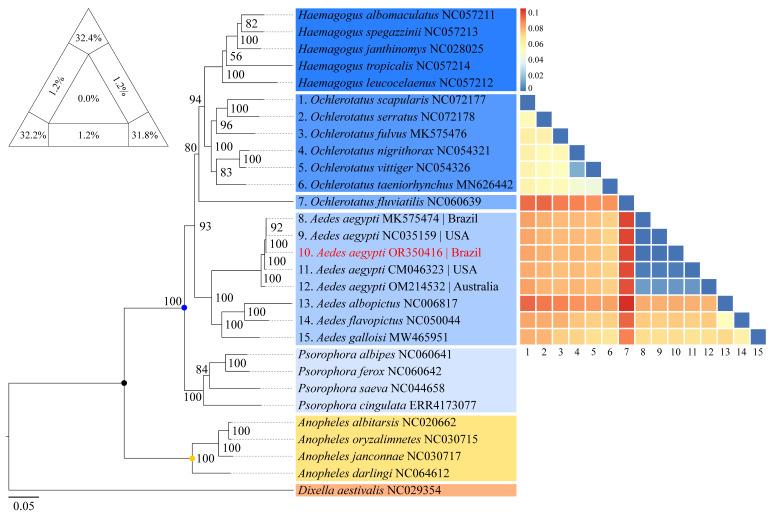
Reconstruction of the phylogeny using the maximum likelihood method of the mosquitoes analysed in the present study, based on the 13 concatenated PCGs of the *Ae. aegypti* obtained in this study and 28 other taxa available in public databases. Support values (BPP) are shown at each node. The coloured dots indicate the taxonomic groups formed in the analysis (the black dot indicates the clade of the Culicidae family; the yellow dot indicates the common ancestor of the Anophelinae subfamily; and the blue circle indicates the common ancestor of the Aedini tribe). The heat map on the right demonstrates the nucleotide distances, based on the maximum composite likelihood method, between taxa related to the genus *Aedes*, where the distances are color coded, ranging from most similar (blue) to most divergent (red). The triangle of values in the upper left corner presents a percentage representation of the quality of the phylogenetic signal for reconstructing the topology based on the set of sequences used. The sum of the sides (96.4%) indicates the percentage of high reliability topologies generated during the analysis.

The sequence obtained in the present study was most similar to those of other *Ae. aegypti* specimens, in particular, sequences with the Genbank IDs MK575476 and NC_035159, with the latter used as a reference for mapping and annotating the DNA structure of the sample analysed here. Based on the analysis of nucleotide distances, the mean pairwise distance recorded among the 29 taxa used to reconstruct the phylogeny, including the sequence obtained here, was 0.09, with values ranging from 0.0005 to 0.1874 (Supplementary Table S3). In accordance with the results of the distance matrix, the phylogeny reconstructed by the maximum likelihood method identified a well-structured monophyletic group with two principal clades, which include representatives of the subfamilies Anophelinae and Culicinae (tribe Aedini), rooted externally by *Dixella aestivalis* (Figure 4). The Culicinae clade is formed four principal sub-clades (*Haemagogus*, *Ochlerotatus*, *Stegomyia* and *Psorophora*), with BPP = 100%, highlighting the taxa most closely related to genus *Aedes* (mean distance of 0.1). The sequence obtained here aligned with the other *Ae. aegypti* (BPP = 100%), as part of the sub-clade of the subgenus *Stegomyia*.

## 4. Discussion

*Ae. aegypti* is now well established as one of the world’s primary vectors of arboviruses, a role favoured by its highly synanthropic behaviour, its desiccation-resistant eggs and its ecological adaptability, which confers a degree of resistance to pesticides, and hamper the implementation of effective control measures [45,46,47]. These traits also contribute to the dispersal capacity of the species and its current worldwide distribution [48,49]. In the 1950s, the implementation of radical measures to control the species in Brazil and other South American countries led to its apparent eradication from the region until its reintroduction in 1970. Since this time, *Ae. aegypti* has become the disease vector with the greatest impact in the region for the transmission of the infectious agents responsible for diseases such as Dengue, Zika and Chikungunya, with thousands of cases being recorded annually, particularly in urban environments [50]. It is in these urban environments that the investigation of the genetic structure of the vector populations can contribute most to the development of the most effective strategies of control. These data not only contribute to the understanding of the adaptive capacity of the vector to different environments, in particular anthropogenic habitats, but also improve the scientific comprehension of the molecular interactions at the pathogen–host level [46,50,51].

The use of molecular markers in studies of the evolutionary biology of mosquitoes, in particular those derived from genomic regions such as the mitochondrial (mtDNA) and nuclear DNA (nDNA), have provided important insights into the evolutionary biology of these insects, as well as the genetic diversity of their populations, in particular in Brazil [46]. In this context, a series of previous studies have concluded that the *Ae. aegypti* populations of Brazil encompass two principal genetic groups, separated approximately by the transition zone at the limit of the Amazon forest [52,53,54,55,56]. The previous studies on populations from Maranhão, in particular, found high levels of intra-population differentiation, which were consistent with the coexistence of two *Ae. aegypti* genetic lineages in this state, which coincides with the ecological transition to the Amazon forest [8,11,57].

The present study describes the complete mitochondrial sequence of an *Ae. aegypti* specimen sampled from one of the populations found in the municipality of Balsas, in the Brazilian state of Maranhão. The sequence obtained is subdivided into 37 functional subunits, in addition to a partial portion of the control region rich in adenine and thymine (A+T), with the characteristic arrangement and disposition of the genes along the two strands of transcription, as observed in all the previous description of the mitochondrial genomes of mosquitoes, in particular, those of the tribe Aedini [20,22,23,24,58]. The nucleotide sequence obtained here share a high level of identity with a reference genome (GenBank ID: NC_035159), which was obtained in a study that also described the three chromosomes of the species [58].

In comparison with the reference genome, the sequence obtained in the present study had a general AT content higher than that of GC, with a positive global skew in AT and negative skew in GC, which is consistent with the larger proportions of adenine and cytosine found throughout the principal sequence. Despite these major similarities, the most obvious difference is the relative lack of polymorphisms, which are most concentrated between the *ND5* and *ND6* regions, in addition to a lack of coverage for the central part of the final portion of the sequence obtained here, which is rich in A+T. In metazoans, this region is associated with the replicative processes of the mtDNA, in particular, a large number of homopolymeric repetitions, considering the presence of significant variations in the length of this region due to the high nucleotide substitution and insertion/deletion rates, which result in clear differences in its length in the different culicid species [14,59,60]. However, in some of the studies that have described mitochondrial sequences by High Throughput Sequencing, certain difficulties arose in the sequencing of this region, possibly related to the problems associated with the recovery of the homopolymeric portions. Given this, the use of selective amplification methods for this region, such as conventional PCR and Sanger sequencing, have been considered to be a potentially valuable alternative, with satisfactory results in some similar studies [61,62,63,64].

The *Ae. aegypti* sequence obtained in the present study contained all 22 tRNAs predicted for the mtDNA, as observed in both other mosquitoes and other organisms, as well as their typical secondary structures, including the substitution of the dihydrouridine (DHU) arm by a DHU loop in tRNA*^Ser1^* gene. With the exception of the 920 bp final portion, in fact, the sequence obtained here includes only 204 non-codifying base pairs, divided among the 22 small intergenic regions, of which the largest was 45 bp, and was located between the tRNA*^Gln^* and tRNA*^Met^* genes, highlighting their compact structure. This has also been observed in other genomes of other mosquitoes, including a degree of overlap between some open read frames (ORFs), in particular those associated with regions involved in the codification of proteins, where, with the exception of only the *ND1* and *ND5* subunits, the start codons are all ATN, while all the stop codons are TAA, in addition to a high AT content in the third codon position in the expressed amino acid triplets. The considerable increase in the AT content at this position may be related to a less intense level of purifying selection against deleterious mutations in these regions [19,65].

In the specific case of the type of selection pressure affecting the PCGs of the sequence obtained in the present study, there was a clear global tendency for the purifying selection (*dN/dS* < 1), which was reflected in the much higher rates of synonymous substitutions, in comparison with non-synonymous substitutions, within the regions analysed. As observed in other studies, in addition, including those of the genus *Aedes* [22,24,65], the purifying selection was more intense in mitochondrial complexes III (cytochrome b) and IV (cytochrome oxidase c), in contrast with complex I (NADH) and the *ATP8* subunit, in particular. While the mean *dN*/*dS* ratio recorded in the present study, considering all the taxa analysed, was relatively low in *ATP6*, the *COI* subunit presented the lowest proportions recorded in the present study, which reinforces the widespread use of this subunit as a molecular marker for the determination of phylogenetic relationships at a given taxonomic level [65,66,67], given its high rates of synonymous mutation in comparison with the regions of complexes I and V (in particular *ATP8*). While the *dN*/*dS* ratio of these regions is still below one, they have less conservative evolutionary restrictions.

Based on the whole concatenated sequence used for the phylogenetic reconstruction, the representatives of the tribe Aedini formed a well-structured monophyletic group that included five sub-clades, of which, two correspond to the genera *Haemagogus* and *Psorophora*, while the other three contain taxa closely related to the genus *Aedes*. It is important to note here that both the arrangement of the taxa and the classification of some of the *Aedes* subgenera have been the subject of intense revision and constant debate, particularly in relation to certain features of the external morphology. In this context, taxa previously considered to be subgenera of *Aedes*, such as *Ochlerotatus* and *Stegomyia*, have been treated as independent groups within the tribe Aedini by some authors, based on the polyphyletic relationships found by studies such as those of Reinert [68] and Reinert et al. [69,70]. However, these reclassifications are not supported universally, given that other studies [71] have not found sufficient support for the independence of the lineages.

The sequence obtained here was most similar to those of the other *Ae. aegypti*, with the clear formation of a sub-clade in *Stegomyia.* The relationships observed here among the *Aedes* sub-clades, considering *Ochlerotatus* and *Stegomyia*, together with *Oc. fluviatilis* (which was classified here as *Georgecraigius fluviatilis* in accordance with the proposal of Reinert [68]), are consistent with the polyphyletic arrangement proposed by this author based on morphological features. These findings also align with those of other studies based on the application of molecular markers to the taxonomy of the tribe Aedini [23,24,72,73].

Three thousand cases of Dengue were recorded in the state of Maranhão in the first half of 2023 alone, with an increase of 30% in the number of probable cases in comparison with the same period of the previous year [74]. This preoccupying trend may be related in particular to the intrinsic deficiencies of the measures adopted for the control and eradication of the populations of *Ae. aegypti*, the principal vector found in urban environments. In this context, it will be necessary for local research groups to evaluate the presence of different lineages of the species, associating this information with the mechanisms of dispersal of the mosquito and the spatial distribution of the outbreaks of Dengue in the state. The first genomic studies in the state of Maranhão focused on the use of mitochondrial (especially *ND4*) and nuclear (microsatellites) molecular markers to describe the genetic structuring of local populations [8,11,12]. Until recently, however, *High Throughput Sequencing* had not been used to determine these genomic features. In this case, through the definition of the complete mitochondrial sequence of *Ae. aegypti*, the present study intended to contribute to the understanding of the evolutionary dynamics of the species, as well as providing resources for its more reliable taxonomic identification, based on molecular features. These advances represent valuable insights for the establishment of guidelines for the development of innovative strategies for the control of this important disease vector.

## Data Availability

All the data obtained during this study are available in the tables and figures included in the text, and in the Supplementary Material. The mitochondrial sequence obtained here was deposited in the GenBank database under accession code OR350416.

## Supplementary Materials

The following supporting information can be downloaded at: https://www.mdpi.com/article/10.3390/insects14120938/s1, Figure S1—Secondary structures of tRNAs and rRNAs of *Aedes aegypti* OR350416; Table S1—Taxa used in the evolutionary analyses; Table S2—*Aedes aegypti* OR350416 nucleotide composition metrics; Table S3—Nucleotide distances by maximum likelihood composition.
