# Supplementary figures and images for: Sequencing and Analysis of the Mitochondrial Genome of Aedes aegypti (Diptera: Culicidae) from the Brazilian Amazon Region
Source: Insects. 2023 Dec 11;14(12):938. doi: 10.3390/insects14120938 (PMC10744036; doi:10.3390/insects14120938)

Supplementary figure 1 - Secondary sctructures of tRNAs and rRNAs of *Aedes aegypti* OR350416

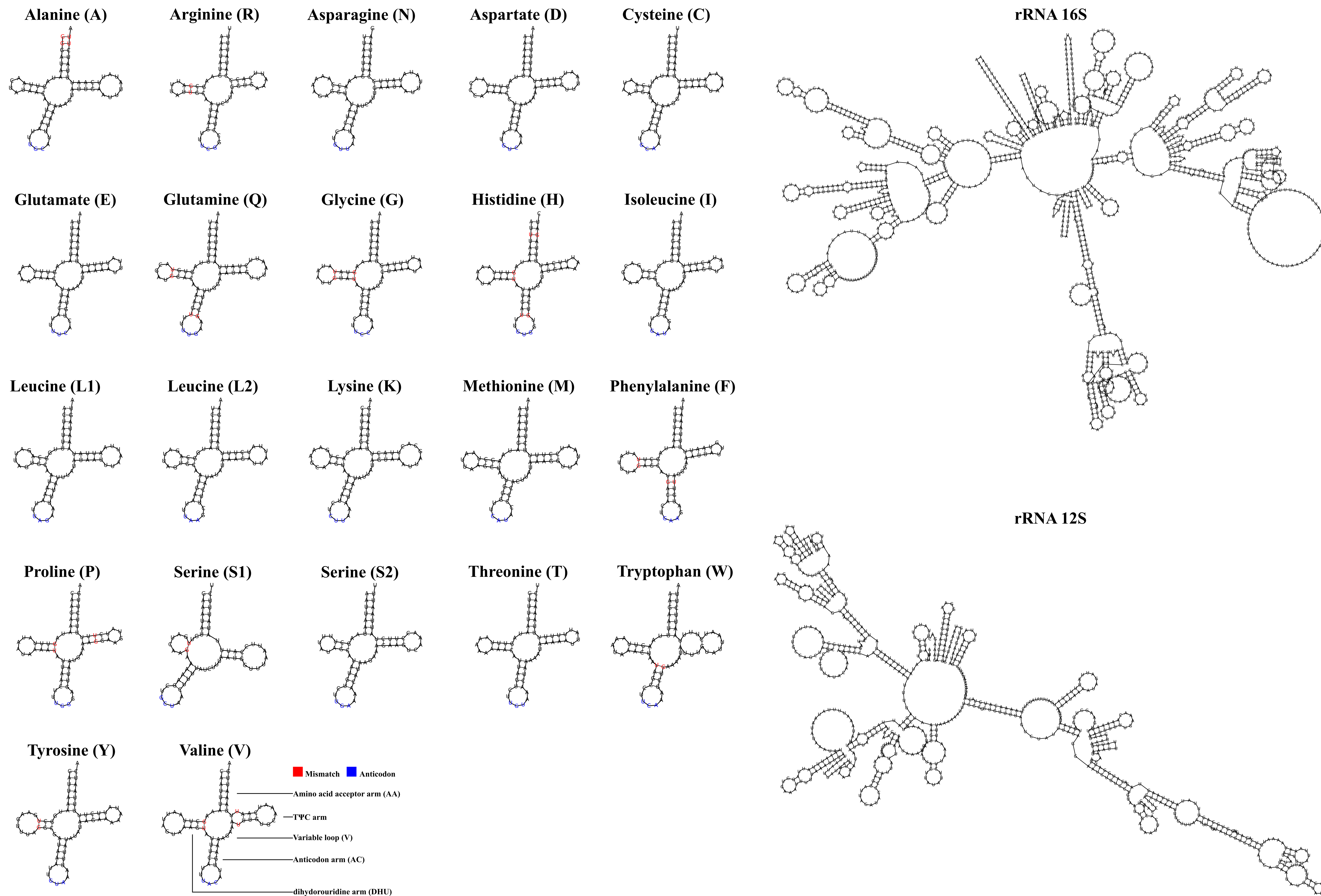

Supplement: Supplementary file 1 [file insects-14-00938-s001.zip › supplementary_figure_1.pdf]
